# Clinical importance of serum heme oxygenase-1 measurement in patients with acute exacerbation of idiopathic pulmonary fibrosis triggered by coronavirus disease 2019

**DOI:** 10.1016/j.rmcr.2022.101615

**Published:** 2022-02-20

**Authors:** Yu Hara, Yume Oshima, Yoichi Tagami, Ayako Aoki, Hiroaki Fujii, Ami Izawa, Kenichi Seki, Akinori Kanai, Aya Yabe, Keisuke Watanabe, Nobuyuki Horita, Nobuaki Kobayashi, Takeshi Kaneko

**Affiliations:** a Department of Pulmonology, Yokohama City University Graduate School of Medicine, Yokohama, Japan; b Department of Pulmonology, Yokohama Minami Kyousai Hospital, Yokohama, Japan

**Keywords:** Biomarker, Coronavirus disease 2019, Heme oxygenase-1, Lung injury burst, Macrophage

## Abstract

A 70-year-old man diagnosed with idiopathic pulmonary fibrosis (IPF) one year earlier developed progressive exertional dyspnea 3 weeks after onset of coronavirus disease 2019 (COVID-19). High-resolution computed tomography showed new extensive ground-glass opacities with rapidly progressive honeycombing. Although he was diagnosed with acute exacerbation (AE) of IPF triggered by COVID-19 and received methylprednisolone pulse therapy twice within one month, there was no improvement of oxygenation and lung involvement. Three months after COVID-19 onset, it was decided to provide best supportive care. An AE of IPF as a sequela of COVID-19, which is recognized as macrophage activation syndrome, is fatal, and in this case, the measurement of serum heme oxygenase-1, which is a macrophage activation biomarker involved in pulmonary cellular protection against oxidative stress, was useful for tracking disease activity.

## Abbreviation lists

AE: acute exacerbation
ARDS: acute respiratory distress syndrome
CARDS: coronavirus disease 2019-associated ARDS acute respiratory distress syndrome
COVID-19: coronavirus disease 2019
DEX: dexamethasone
GGO: ground-glass opacity
HO: heme oxygenase
HRCT: high-resolution computed tomography
IP: interstitial pneumonia
IPF: idiopathic pulmonary fibrosis
KL: Krebs von den Lungen
LDH: lactate dehydrogenase
mPSL: methylprednisolone
PSL: prednisolone
SARS-CoV-2: severe acute respiratory syndrome coronavirus-2

## 1 Introduction

Acute exacerbation (AE) is the main cause of death in the natural course of idiopathic pulmonary fibrosis (IPF), in which the histological pattern typically involves diffuse alveolar damage superimposed upon lung fibrosis. Severe acute respiratory syndrome coronavirus-2 (SARS-CoV-2) has been considered a particularly likely cause of AE, based on the similarities in the clinical, radiological, and pathological presentation between AE of IPF and viral pneumonitis. Patients with IPF have been reported to be at increased risk of death from coronavirus disease 2019 (COVID-19), particularly those with poor lung function and obesity, but there have been few reports discussing the detailed clinical course of AE of IPF triggered by COVID-19 [1].

Macrophage polarization plays key roles in all phases of wound healing, which are inflammation, proliferation, and remodelling (fibrosis), and the interaction between M1 and M2 macrophages derived from peripheral monocytes (uncommitted macrophages (M0)) is reported to be closely correlated with disease progression in patients with AE of IPF and CARDS (COVID-19-associated acute respiratory distress syndrome (ARDS)) [2,3]. Heme oxygenase (HO)-1 is a 32 kDa heat shock protein that converts heme into carbon monoxide (CO), free iron (Fe^2+^), and biliverdin (bilirubin) [4]. The HO-1 system with these products represents a powerful tissue protective system, and it regulates important biological processes, including inflammation, antioxidant status, apoptosis, cell proliferation, fibrosis, and angiogenesis. HO-1 is expressed exclusively on anti-inflammatory M2 macrophage linage, but not pro-inflammatory M1 macrophage by heat shock and oxidative stress conditions and the anti-inflammatory and anti-oxidative actions of each product originating from HO-1, CO and biliverdin (bilirubin), sustain the properties of M2 [3,5]. The prior research suggested that serum HO-1 measurement increased mainly in alveolar macrophages of patients with AE of interstitial pneumonia (IP) reflecting protection against oxidative stress and activated M2 macrophages and contributed to detect AE and predict disease prognosis in patients with IP [6,7].

A case of AE of IPF triggered by COVID-19, in which serum HO-1 measurement was considered useful for tracking the disease activity of lung injury burst, is presented.

## 2 Case presentation

**Fig. 1:**
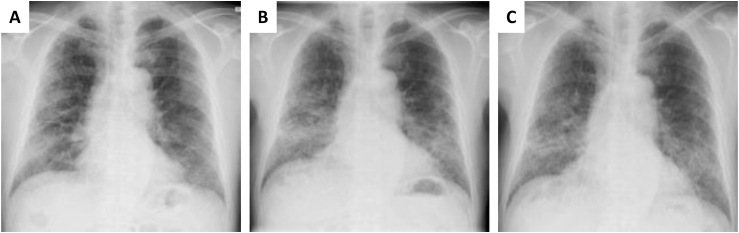
Serial changes of chest radiograph findings after the diagnosis of coronavirus disease 2019 (COVID-19). (A) At the diagnosis of COVID-19 (B) At the diagnosis of acute exacerbation of IPF (C) After the first methylprednisolone pulse therapy.

**Fig. 2:**
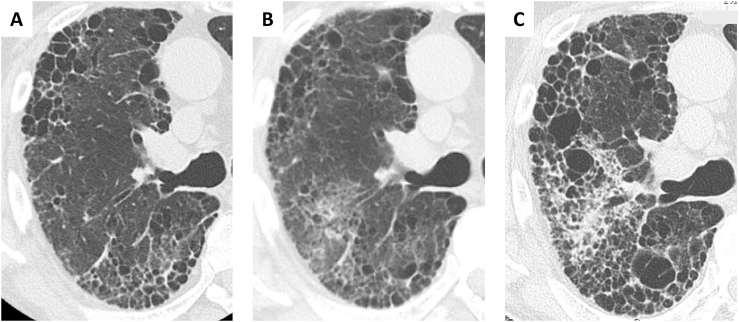
Serial changes of high-resolution CT findings before the diagnosis of acute exacerbation (AE) of idiopathic pulmonary fibrosis (IPF). (A) At the diagnosis of IPF (B) At the diagnosis of coronavirus disease 2019 (C) At the diagnosis of AE of IPF.

**Fig. 3:**
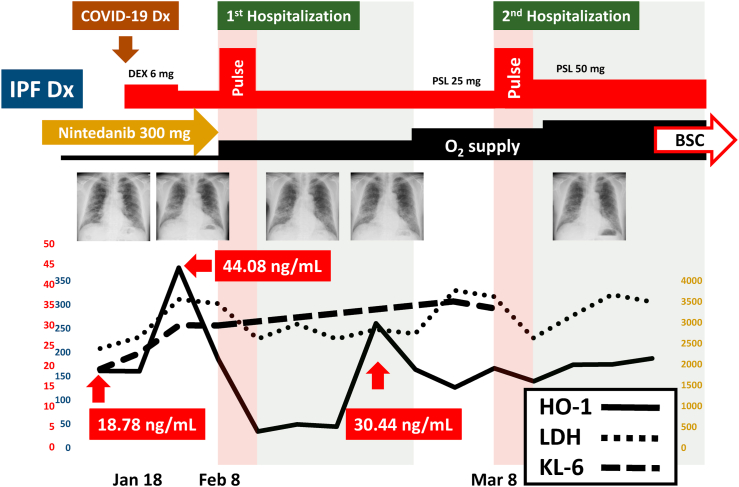
Clinical course. Abbreviation lists: BSC, best supportive care; DEX, dexamethasone, HO, heme oxygenase; IPF, idiopathic pulmonary fibrosis; KL-6, Krebs von den Lungen-6; LDH, lactate dehydrogenase; PSL prednisolone.

A 70-year-old man who started nintedanib 1 year earlier with a diagnosis of IPF based on typical usual interstitial pneumonia pattern of high-resolution computed tomography (HRCT) developed progressive exertional dyspnoea 1 week after 10-day dexamethasone (6 mg/day intravenously) treatment for COVID-19. Fine crackles were heard in the chest bilaterally. Blood tests at the time of hospitalization showed marked increases of serum HO-1 (44.8 ng/mL (normal control: 9.43 ng/mL), lactate dehydrogenase (LDH) (302 U/L (RI: 124–222 U/L)), and Krebs von den Lungen (KL)-6 (2931 U/mL (RI: ≤500 U/mL)). The bilateral extensive ground glass opacity (GGO) with lung volume loss in the basal fields newly appeared (Fig. 1A and B). Also, comparing the HRCT findings showing honeycombing with subpleural predominance of both basal lungs 1 year earlier, peribronchiolar patchy GGO appeared bilaterally at COVID-19 onset (Fig. 2A and B) and marked progression of honeycombing with increased intensity of GGO lesions was observed at the time of diagnosis of AE that developed 3 weeks later (Fig. 2C). Methylprednisolone (mPSL) pulse therapy for 3 days (1000 mg/day intravenously) was initiated under the diagnosis of AE of IPF triggered by COVID-19 and corticosteroid (oral prednisolone) were maintained at 25 mg/day (body weight 70 kg) rather than tapering to prevent the deterioration of type II diabetes as his comorbidity. After the mPSL pulse therapy, high HO-1 and LDH decreased with the improvement of chest radiograph findings (Fig. 1C), however, about 1 month after the first mPSL pulse therapy, progressive dyspnea and desaturation were observed with re-elevation of HO-1 and LDH. Against the second mPSL pulse therapy for re-exacerbation, the decrease in HO-1 was limited compared to the first pulse, and KL-6 showed persistently increased. In summary, although he received mPSL pulse therapy twice within one month for the diagnosis of AE, there was no improvement of oxygenation and lung involvement. Three months after COVID-19 onset, we switched the treatment strategy to best supportive care with oral corticosteroid maintenance therapy (50 mg/day) (Fig. 3).

## 3 Discussion

We experienced the case of IPF that developed a steroid pulse-resistant AE about 3 weeks after the onset of COVID-19 with remarkably rapid progression of fibrosis. Also, the changes of serum HO-1 as an M2 macrophage activation biomarker and the correlation with other blood biomarkers for IPF including LDH and KL-6 were examined and we found that the serial changes of serum HO-1 might reflect disease activity of AE unlike carboxyhemoglobin and bilirubin which were metabolites of HO-1. (Yokohama City University institutional review board number: B170900025). The details of the enzyme-linked immuno sorbent assay method have been described previously [8]. The median serum HO-1 levels for normal control subjects included 45 healthy, non-smoking adults who had been admitted to the hospital for a medical check-up were 9.43 ng/mL and 63 patients with idiopathic interstitial pneumonia at the stable state were 16.3 ng/mL (data not shown).

Macrophages/monocytes are critical contributors to innate immune responses in COVID-19 [2]. The excess production of inflammatory cytokines, known as cytokine storm, in which proliferation and activation of macrophages/monocytes are driven, is thought to be a major cause of disease progression in COVID-19. Inhaled SARS-CoV-2 binds angiotensin-converting enzyme 2-expressing type II pneumocytes, triggering the immune-responses-induced the oxidative stress and leading to the lung damage [9,10]. The type II pneumocytes reside the alveolar walls and alveolar macrophages in its sacs, which occupy more than 90% of resident immune cells [11]. Macrophages are broadly divisible into two groups: pro-inflammatory, classically activated macrophages (M1) and anti-inflammatory, alternately activated macrophages (M2). It has been recently clarified that SARS-CoV-2 distinctively infects M1, but not M2 [12]. The polarization of macrophage is very critical factor for the pathogenesis of COVID-19. HO-1 is a protein whose expression is strongly and exclusively induced on the anti-inflammatory M2 macrophage lineage, but not pro-inflammatory M1 macrophages by heat shock and oxidative stress conditions [3]. HO-1 is increased mainly in alveolar macrophages of patients with various pulmonary diseases such as ARDS and AE of IPF, reflecting the activation of M2 macrophages against oxidative stress, which contributes to progressive lung fibrosis by producing large amounts of transforming growth factor, resulting in extracellular matrix deposition, epithelial-mesenchymal transition, fibroblast activation, and cell death [6].

Serum HO-1 measurement was reported to be useful to assess disease activity and predict treatment outcomes in patients with ARDS and AE of IPF [7]. Consistent with the previous research, in this case diagnosed with AE of IPF triggered by COVID-19, serum HO-1 at the diagnosis of both first AE and second AE increased (first AE: 44.08 ng/mL, second AE: 30.44 ng/mL) than that at the diagnosis of IPF (18.78 ng/mL) and the serial changes of serum HO-1 levels seemed to reflect disease activity of AE. However, there was a discrepancy between the serum HO-1 increase and serum LDH increase or initiation of steroid pulse therapy in clinical practice (Fig. 3). This phenomenon could be explained because the serum HO-1 increase, which reflected the activity of M2 macrophages due to the cytoprotective reaction on oxidative stress, occurred before the serum LDH increase which reflected the actual destruction of lung cells or initiation of steroid pulse therapy. Furthermore, the volatility of serum HO-1 (135%) calculated from the baseline (18.78 ng/mL) and first AE diagnosis (44.08 ng/mL) was greater than that of serum LDH (45%) calculated from the same timing (baseline LDH: 208 U/L, first AE diagnosis: 302 U/L). Serum KL-6 which was a high-molecular-weight mucin-like glycoprotein, known as human mucin-1 was thought not to be suitable for tracking the disease activity of lung injury because of low volatility [13]. Therefore, it is very important to immediately start adequate anti-inflammatory treatment by monitoring the serial changes of serum HO-1 and prevent the rapid progression of lung fibrosis observed in this case, because pulmonary HO-1 expression as a biomarker of M2 activation contributes to progressive end-stage fibrosis [3,6].

## 4 Conclusion

Serum HO-1 reflecting M2 macrophage activation could be clinically useful for tracking severity and predicting prognosis of patients with AE of IPF triggered by COVID-19. Controlling the activation of M2 macrophages immediately and sufficiently might be the therapeutic target for the lung injury burst. In addition, the disease mechanism of this case was AE of IPF limited to the lung, not CARDS with systemic inflammation (cytokine storm). Because as previously research, serum HO-1 in ARDS is higher than AE of IPF, we speculated that the HO-1 is abundantly produced from reticuloendothelial tissues macrophages such as spleen, liver, and bone marrow in CARDS [7]. Therefore, serum HO-1 measurement could contribute to the distinction between CARDS and AE of IP.

## Statement confirming consent

Appropriate written informed consent was obtained for publication of this case report and accompanying images.

## Funding statement

This research was supported by the Yokohama City University Research Fund.

## Declaration of competing interest

None of the authors have any conflicts of interest to declare.
